# Early origins of allergy and asthma (ARIES): study protocol for a prospective prenatal birth cohort in Chile

**DOI:** 10.1186/s12887-020-02077-x

**Published:** 2020-04-15

**Authors:** Caroll D. Hernández, Paola Casanello, Paul R. Harris, José A. Castro-Rodríguez, Carolina Iturriaga, Guillermo Perez-Mateluna, Marcelo Farías, Marcela Urzúa, Cherie Hernandez, Carolina Serrano, Mauricio Sandoval, Rodrigo Hoyos-Bachiloglu, Ricardo Uauy, Arturo Borzutzky

**Affiliations:** 1 Department of Pediatric Infectious Diseases and Immunology, School of Medicine, Pontificia Universidad Católica de Chile, Diagonal Paraguay 362, 8th floor, 8330077 Santiago, Chile; 2 Department of Neonatology, School of Medicine, Pontificia Universidad Católica de Chile, Santiago, Chile; 3 Department of Obstetrics, School of Medicine, Pontificia Universidad Católica de Chile, Santiago, Chile; 4 Department of Pediatric Gastroenterology and Nutrition, School of Medicine, Pontificia Universidad Católica de Chile, Santiago, Chile; 5 Department of Pediatric Pulmonology and Cardiology, School of Medicine, Pontificia Universidad Católica de Chile, Santiago, Chile; 6 Unidad de Nutrición Básica, Instituto de Nutrición y Tecnología, Instituto de Nutrición y, Tecnología de los Alimentos (INTA), Universidad de Chile, Santiago, Chile; 7 Millennium Institute on Immunology and Immunotherapy, School of Medicine, Pontificia Universidad Católica de Chile, Santiago, Chile

**Keywords:** Birth cohort, Cohort study, Allergy, Asthma, Prenatal conditions, Food allergy, Atopic dermatitis

## Abstract

**Background:**

Growing evidence shows that atopic dermatitis (AD), food allergy (FA), allergic rhinitis, and asthma are largely determined during the first 1000 days (time elapsed from conception to the 2nd birthday). The ARIES birth cohort aims to determine prenatal and perinatal conditions, as well as genetic and epigenetic factors, that participate in the early setting of immune responses, and the role of these in the later determination of the risk of allergic diseases and asthma in the offspring.

**Methods:**

We have designed a birth cohort of 250 families with prenatal recruitment (~ 14 weeks). We will genotype relevant allergy/asthma-associated variants in trios and will perform immunophenotyping and evaluation of allergy biomarkers in cord blood. At 1 and 2 years of age we will assess if infants have developed allergic sensitization, AD, FA, as well as biomarkers of asthma including the asthma predictive index. We will also evaluate how maternal conditions modify immune programming through epigenetic modifications and will then depict newborn epigenetic cues of allergy/asthma risk. Next, we will assess composition/diversity of maternal gut, placenta, breastmilk and infant gut microbiome and their association with immunophenotype and biomarkers at birth, and clinical outcomes at age 1 and 2. Finally, we plan to assess how environmental exposures (perinatal outdoor and indoor pollution, allergens and endotoxin) affect the incidence of allergic sensitization, AD, FA, and risk of asthma.

**Discussion:**

The in-depth study of the ARIES birth cohort shall provide crucial information to understand the rising incidence of allergies and asthma in developing countries, and hopefully provide cues on how to prevent and treat these diseases.

**Trial registration:**

clinicaltrials.gov NCT04186949, retrospectively registered on December 5, 2019.

## Background

The first 1000 days of life of a child (from conception until the second birthday) are crucial in the growth and development of the immune system. Allergic diseases are currently among the most common chronic diseases in the world [1], with rising incidence in developing and developed countries over the last few decades [2–4]. The most prevalent allergic diseases, atopic dermatitis (AD), food allergy (FA), allergic rhinitis (AR), and asthma, mostly begin in early childhood through a process known as the atopic march, which is characterized by a stereotyped progression from AD and food allergy to asthma and AR [5]. Although these diseases are closely intertwined, they are influenced by different genetic and environmental factors that drive each disease, all of which challenge the research of their etiology and pathogenesis.

In Chile, our group and other researchers have recently estimated the prevalence of allergic diseases and asthma to be at 13% for AD [6], 5.5% for FA [6], 21% for AR [7], and 14% for asthma [8]. It is well known that children with early-onset, severe, persistent AD, and elevated levels of total and specific immunoglobulin E (IgE) antibodies, have an increased risk of developing AR and asthma later in life [9, 10]. Moreover, the early sensitization to foods or aeroallergens in the first year of life increases the risk of persistent AD and asthma [11, 12]. Due to the early onset of allergies in many patients, growing evidence shows that these are determined at earlier ages by strong environmental risk factors in genetically susceptible hosts. However, there still is a poor understanding of what shapes the appearance of allergic diseases and asthma in early life.

The cornerstone of the pathogenesis of most allergic diseases is the predisposition to skewed type 2 immune responses and an excessive production of IgE against common allergens, generally known as atopy [13]. Specifically, atopic diseases are characterized by increased type 2 immunity [14], defined by the production of prototypical cytokines interleukin-4 (IL-4), IL-5, IL-9 and IL-13. Through these mediators, type 2 immunity induces an inflammatory response involving innate and adaptive immunity, which is characterized by the participation of eosinophils, mast cells, basophils, type 2 innate lymphoid cells (ILC2), IL-4- and/or IL-13-conditioned macrophages, and T helper 2 (Th2) cells [15]. The dysregulation and activation of Th2-mediated immunity then drives B cells to produce (IgE) antibodies against common food and aeroallergens, which later elicit the clinical symptoms of allergy.

**Fig. 1:**
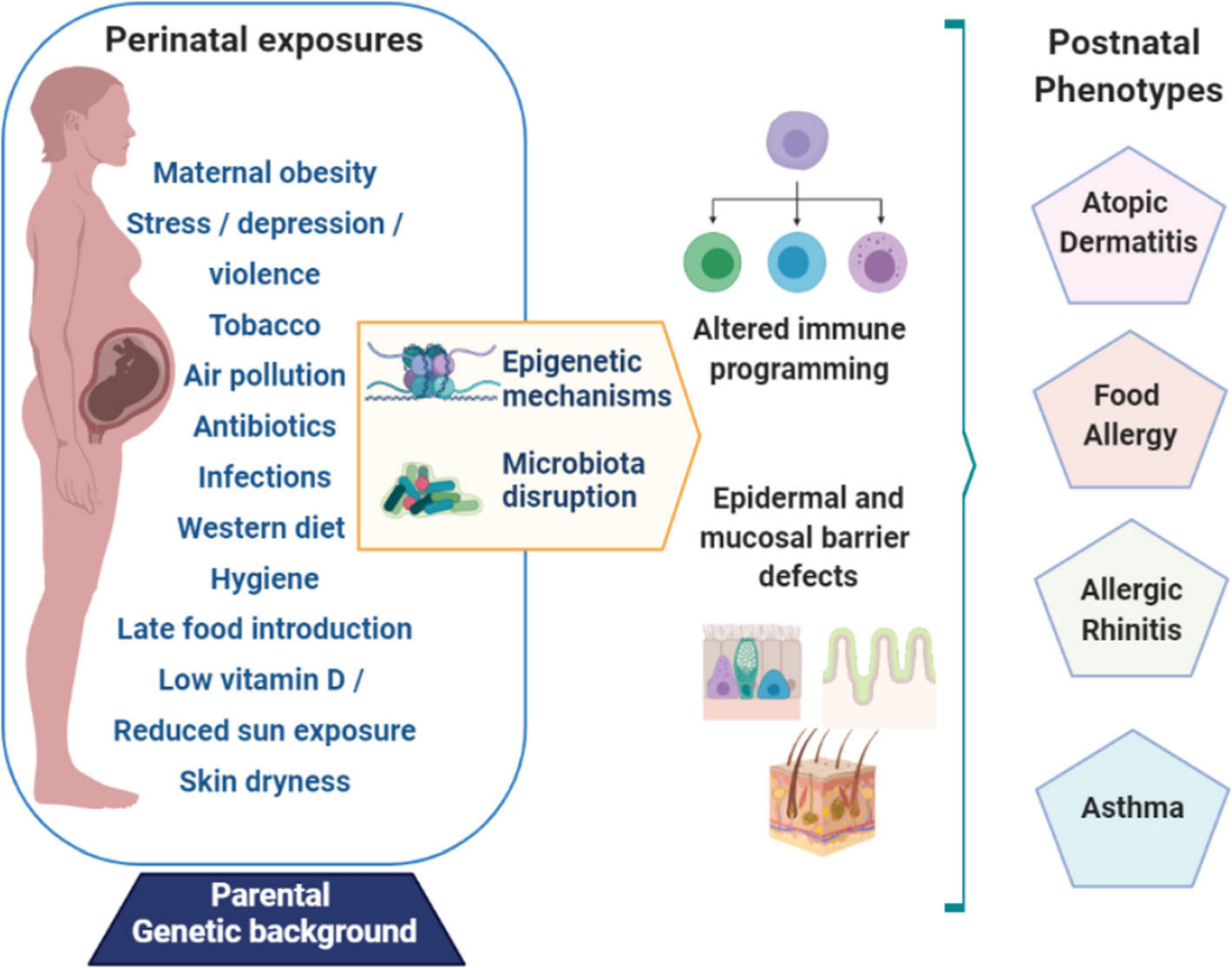
Factors that influence the development of allergy and asthma. Diagrammatic representation of the hypothesis of the ARIES study

Allergic diseases are the result of a complex gene-environment interactions (Fig. 1). In children, the genetic background is an important factor in the development of allergy and asthma, as is demonstrated in twin studies that show a heritability of about 80% for AD [16] and 60% for asthma [17]. Genome-Wide Association Studies (GWAS) have been a successful tool in recent decades to identify multiple genetic variants that influence the susceptibility to develop allergy and asthma [18], but the genome alone cannot fully explain the rising incidence of allergic diseases worldwide. Multiple studies have indicated that epigenetic mechanisms including DNA methylation, histone posttranslational modifications, and micro-RNA-mediated gene expression in immune cells might play an important role in the development of allergic diseases [19].

The microbiota also seems to be key in the development of allergy and asthma in early childhood, as has been suggested in multiple studies of gut, airway, and skin microbiota [20–22]. Moreover, alterations of the microbiota can even precede development of allergies emphasizing a pathogenic role of dysbiosis. For example, studies of gut microbiome samples obtained from infants has demonstrated that a low gut microbial diversity during the first month of life precedes the development of AD at 2 years and asthma at 7 years of age [23, 24].

Finally multiple environmental factors, nowadays encompassed under the term ‘exposome’, appear to have direct or indirect influence on the generation of allergies and asthma. Most noteworthy among these factors are exposure to tobacco smoke and air pollution, decreased sunlight and vitamin D deficiency, obesity, environmental conditions, maternal stress and pregnancy complications, prematurity, and timing of food and aeroallergen exposure [25]. However, a myriad of other exposures that are still matter of study worldwide probably may influence allergy and asthma risk.

Altogether, genetic and epigenetic factors strongly interact with the environment starting in utero and then throughout early childhood to shape the risk, incidence, and severity of allergic diseases and asthma. However, many aspects of these interactions are poorly understood, making it difficult to envision the global picture of gene-environment interactions determining allergic diseases and asthma, as well as the pathogenic mechanisms behind these. To delve into these questions, we have designed the “eArly oRIgins of allErgy and aSthma” (ARIES) birth cohort that seeks to study and understand how the interaction of genetic factors, maternal conditions, maternal and child microbiome, and the environment, epigenetically shape the immune programming in the first 1000 days of life to determine the development of allergic diseases and asthma.

## Study objectives

### General objective

To determine prenatal and perinatal conditions, as well as genetic and epigenetic factors, that participate in the early setting of immune responses, and the role of these in the later determination of the risk of allergic diseases and asthma in the offspring.

### Specific objectives

1. To determine the early programming of the immune system by determining genotype, immunophenotype, and allergy biomarkers in cord blood of neonates at high-risk for allergic diseases/asthma and controls at low risk for allergic diseases and asthma.
2. To evaluate how maternal conditions (i.e. obesity, metabolic, inflammatory status) modify the immune programming and generation of immune tolerance through epigenetic modifications.
3. To evaluate the composition and diversity of maternal gut, placenta, breastmilk and newborn/infant gut microbiome and their association with the immunophenotype and allergy biomarkers at birth.
4. To assess how environmental exposures during the first 1000 days affect the incidence of allergic sensitization, AD, FA, and risk of asthma.

## Methods

### Study design and setting

The ARIES birth cohort is an observational prospective longitudinal study carried out in Santiago, Chile, at the UC Christus Health Network. UC Christus is a private health network associated to the School of Medicine of the Pontificia Universidad Católica de Chile. Its hospitals attend nearly 2200 deliveries per year, mostly from Santiago’s central and urban communes. The study aims to include 250 families, from which the pregnant women are recruited during pregnancy, with emphasis in incorporating women with early pregnancies. The study includes obtaining maternal, paternal, and infant clinical history and biological samples, in addition to an in-depth evaluation of indoor air contaminants and dust samples during pregnancy and infancy. This study has been designed following the guidelines of the STrengthening the Reporting of OBservational studies in Epidemiology (STROBE) statement [26].

### Recruitment of study subjects

Invitations for participation are being extended through leaflets, videos, and/or personal interviews to pregnant women with at least 8 weeks of gestation, who schedule their delivery at the UC Christus health network hospitals and outpatient centers. We deliberately avoid excluding mothers with multiple pregnancy, pathological conditions, or pregnancy complications, in order to include a wide spectrum of conditions that may affect development of allergies and asthma. Similarly, we will not exclude preterm deliveries or infants born with neonatal complications to reduce any bias that could result from restricting sampling to normal pregnancies and children. The biological father of every ARIES newborn is also invited to participate. All participant parents will be given detailed information about the study and will be requested to sign informed consent forms. Each subject will be assigned a study ID number.

### Data collection

The pregnant women undergo a clinical assessment at different time-points, according to the cohort timeline (Table 1). Collected data include demographics, socioeconomic information, education level, lifestyle, nutrition, environmental, physical and mental health, household and family information. The maternal antenatal visits also include recording of maternal anthropometric data, medical history of diseases, diet, and use of medications and nutritional supplements. Study data is being collected and managed using REDCap (Research Electronic Data Capture) electronic data capture tools hosted at Pontificia Universidad Católica de Chile [27].

**Table 1:** Overview of data and sample collection in the ARIES birth cohort

	Mother		Father	Child	Mother		Child	
	Recruitment 8–20 and 24–30 weeks	Delivery	8–42 weeks	Pre-discharge	3 months Post-partum	6 months Post-partum	1 and 2 years	1 and 2 years
*Medical history and anthropometry*	**X**	**X**	**X**	**X**	**X**	**X**	**X**	**X**
*Medicines used*	**X**	**X**	**X**	**X**	**X**	**X**	**X**	**X**
*Physical exam*	**X**			**X**			**X**	**X**
*Allergy questionnaires*	**X**		**X**				**X**	**X**
*Lifestyle habits questionnaires*	**X**		**X**				**X**	**X**
*Depression/stress scales*	**X**				**X**	**X**		
*Blood samples*	**X**	**X**	**X**				**X**	**X**
*Hair sample*		**X**		**X**				
*Cord blood samples*		**X**						
*Saliva sample*	**X**						**X**	**X**
*Stool microbiota sample*	**X**			**X**			**X**	**X**
*Breastmilk microbiota sample*		**X**						
*Skin microbiota sample*	**X**			**X**			**X**	**X**
*Vaginal microbiota sample*	**X**							
*Placenta samples*		**X**						
*Oral microbiota sample*				**X**			**X**	**X**
*Nasal microbiota sample*				**X**			**X**	**X**
*Indoor air quality measurements*	**X**				**X**			
*Indoor house dust samples*	**X**				**X**			

### Questionnaires and surveys

Before delivery, we are applying detailed surveys to the family regarding allergies to determine the presence of AD, FA, AR, and asthma in parents and siblings. Additionally, we are asking about the indoor and outdoor environment where the parents live, their lifestyle habits (e.g. smoking), and dietary habits (Table 2).

**Table 2:** Questionnaires of the ARIES birth cohort

Questionnaires	Description
**Perceived Stress Scale (PSS14)**	This scale is a self-report instrument that evaluates the level of stress perceived during the last month. It has 14 items, the responses have a five-point scale (0 = never, 1 = almost never, 2 = occasionally when, 3 = often, 4 = very often). The score obtained indicates that a higher score corresponds to a higher level of perceived stress.
**Edinburgh Postnatal Depression Scale (EPDS)**	This scale consists of ten short statements. The mother chooses which of the four possible answers is the one that most closely resembles the way she felt during the previous week. The answers have a score of 0, 1, 2 and 3. The total score is calculated by adding the scores for each of the 10 items. During the pregnancy, a score of 13 or more points indicates suspicion of depression. In postpartum, a score of 10 or more points indicates suspicion of postpartum depression. If the statement “I had the idea of hurting myself” is not answered as ‘never’, the patient requires additional mental health evaluation within 24 h.
**Allergy and asthma surveys**	Detailed description of allergy history and symptoms, physician diagnosis, and medical treatment, to determine diagnosis of atopic dermatitis, food allergy, asthma, and allergic rhinitis
**Environment and habits**	Detailed survey regarding type of residence and housing, pollution, heating, home cleaning habits, and pets. Habits survey asks about drug use, alcohol consumption, tobacco smoking habits, physical activity, time spent outdoors and using TV and/or other screens.
**Food frequency questionnaire**	Record consumption of vegetables, legumes, types of cereals, low fat meats, high fat meats, dairy, and olive oil.

Between 24 and 30 weeks of pregnancy, and at 3 to 6 months after birth we apply the Edinburgh Postnatal Depression Scale (EPDS) to evaluate maternal depression and the Perceived Stress Scale (PSS-14) to determine levels of stress or anxiety. With these tools, we expect to evaluate the maternal psychological status and how this can affect the child’s health (Table 2).

### Biological samples collection

**Fig. 2:**
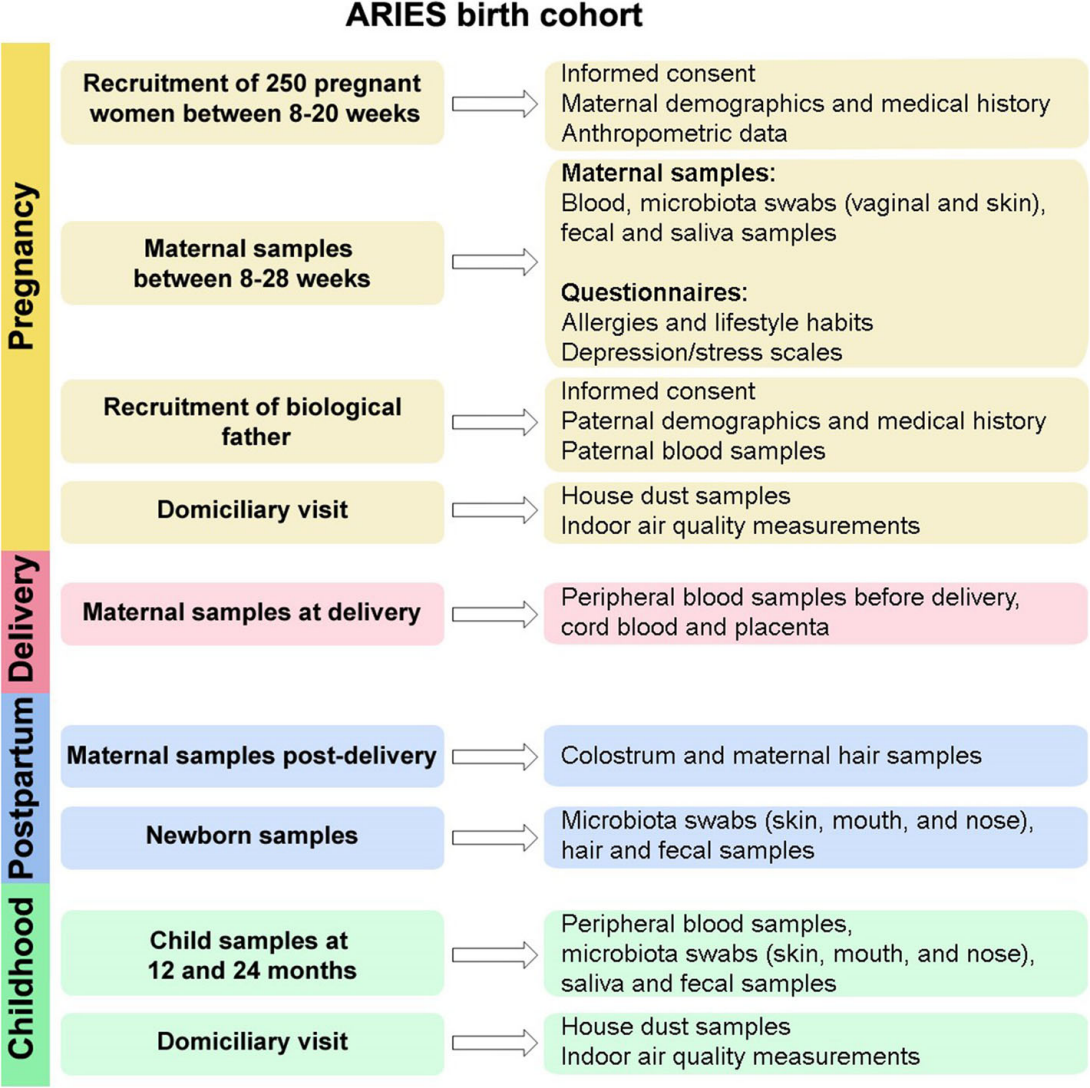
Recruitment and follow-up strategy used in the ARIES birth cohort. Patients that fulfill inclusion criteria will be invited to participate in their 1st antenatal visit and enter the study at 14 ± 6 weeks of gestation with follow-up until the child is 2 years old

Mothers and infants participating in the ARIES cohort will be serially followed from pregnancy until 2 years of age with both questionnaires and biological sample collection (Fig. 2). Biological samples from the mothers are taken at 8–20 weeks, 24–30 weeks, in pre-partum, delivery, and post-partum. Biological fathers will fill out questionnaires and provide one blood sample at any time during the study. All children will be followed with clinical examination and biological samples at birth, 12 months, and 24 months of age. Samples that will not be processed immediately through clinical laboratories will be biobanked in − 80 °C freezers at institutional facilities.

### Blood

Blood samples collected from the parents include blood in vacutainer tube for serum extraction (10 ml), blood in vacutainer EDTA tube for plasma and red blood cell extraction (4 ml), and whole blood for genomic DNA isolate (all from Becton Dickinson).

Additionally, maternal blood samples are taken for routine laboratory tests during pregnancy, including complete blood counts with eosinophil count, HIV, thyroid stimulating hormone, blood ABO-Rh group, fasting glucose, insulin, lipid profile, IgE levels, and the Phadiatop™ test to detect aeroallergen sensitization.

At birth, cord blood is collected from the umbilical cord into vacutainer tubes for serum extraction (2 ml), vacutainer EDTA tubes for plasma and red blood cells extraction (2 ml) and whole blood for DNA isolate (all from Becton Dickinson). Additionally, cord blood is collected for laboratory tests such as complete blood counts, glucose, insulin, lipid profile and IgE levels. The remaining cord blood is collected in a Terumo blood collection bag for cord blood mononuclear cells (CBMC) isolation by Ficoll gradient centrifugation.

At 12 and 24 months, peripheral blood will be obtained from the infants by study nurses, into a vacutainer tube (4 ml) and vacutainer EDTA tube (2 ml) for serum and plasma extraction, respectively. An additional vacutainer sodium heparin tube (10 ml) will be obtained for peripheral blood mononuclear cell (PBMC) isolation by Ficoll gradient centrifugation.

The serum and plasma samples will be used to evaluate biomarkers of allergy and asthma determination. The genomic DNA isolated from whole blood will be used for genotyping studies, while the mononuclear cells (CBMC and PBMC) will be used for immunophenotyping.

### Placenta

After delivery, the placenta is examined according to standard operating procedures by the delivery personnel and placed in a plastic bag and stored at 4 °C. Then, the placenta is processed at the laboratory under sterile conditions. Placenta samples are taken for microbiota determination, molecular analysis (DNA, RNA, protein and fatty acids) and environmental contaminants analysis. All the samples are stored at − 80 °C until their use.

### Saliva

Between 0.5 and 3 ml of saliva is collected from the mothers at 8 to 20 weeks of pregnancy, by spitting into a Salivette plastic tube (Sarstedt) for cotinine determination. Additionally, maternal saliva is collected between 24 to 30 weeks of pregnancy, into a Salivette cortisol plastic tube (Sarstedt) for cortisol determination. The tubes are centrifuged (2000 rpm, 10 min) according to manufacturer’s instructions. The eluted saliva is collected into a 1.5 ml tube and frozen at − 80 °C.

At 12 and 24 months of age, infant saliva (around 0.5 ml) will be collected from the buccal cavity using a plastic Pasteur pipette for cotinine determination.

### Vaginal, skin, mouth, and nose swabs

Both vaginal and skin maternal samples are taken with swabs and are collected in 1 ml liquid amies medium in a skirted tube (eSwab, Copan). These samples are obtained between 24 and 30 weeks of pregnancy. In the infant, a skin, mouth, and nose sample are taken using a swab (eSwab, Copan) at birth and at 12 and 24 months of age. All the samples are stored at − 80 °C until their use for microbiome determination.

### Stool

Maternal stool samples are obtained between 24 and 30 weeks of pregnancy at home, into sterile feces containers with an ad-hoc spoon (Sarstedt). The samples are stored in the freezer as soon as possible and then transported to the research laboratory. Participants will be instructed to keep the samples in a cooling bag to prevent thawing of the samples during transport.

Meconium samples are collected from the infant’s nappy by the parents or the study nurse in the nursery and saved in sterile feces containers using an ad-hoc spoon (Sarstedt). At 12 and 24 months of life an infant fecal sample will be collected in the same manner. All the samples will be stored at − 80° until microbiome analysis.

### Breast milk

One or two days after delivery, during postpartum rest, a sample of colostrum is obtained by the mother or by the study nurse and preserved in a sterile tube. The samples are aliquoted and stored at − 80 °C until their use for microbiome determination.

### Infant buccal swab

A buccal swab (FLOQSwabs, Copan) is rubbed on the inside of the infant’s cheeks, stored at − 20 °C until genomic DNA isolation for genotyping studies.

### Hair specimens

Hair specimens are obtained from both mother and newborn. The samples are cut by stainless steel scissors from the occipital part of the head, as close to the scalp as possible. These samples will be used for contaminant determination.

### Handling, transport and storage of biological samples

All the biological samples are transported to the Translational Allergy and Immunology Laboratory, where the samples are processed, aliquoted in small volumes, and stored frozen at − 80 °C. All the samples of each participant are transported to the laboratory with their respective transportation form, identifying sampling time and date, as well as who obtained, transported, and received the sample.

This project contemplates a large number of biological samples, all of which are stored according to sample type (blood, serum, plasma, DNA, saliva, etc), and the source of sample (ID number). Each sample is registered in an electronic registry using the software eLABJOURNAL (BIO-ITECH).

### Assessment of environmental exposures

#### Outdoor air pollution

We will use data provided by citywide local monitoring systems for airborne contaminants belonging to the Chilean Ministry of Health, through which we will evaluate the main air contaminants such as PM 2.5, PM10, and quality of air levels by gases CO, NO_2_, O_2_, SO_2_, O_3_, registered in the nearest monitoring station to the place of residence of each family.

#### Indoor air pollution

Indoor contaminants will be measured in the families’ homes at two different timepoints through home visits. The first measurement will be done between the 24–30 weeks of pregnancy and the second will be done at 3 to 6 months after delivery. Indoor air contaminants CO, CO_2_, O_3_, and NO_2_ will be measured with an indoor air quality detector, model DIAQ100 (JSTEC). This device is placed in the mother and/or infant’s bedroom for 3 to 7 days after which data is extracted and stored.

#### Indoor allergens and endotoxin levels

We will quantify indoor allergens and endotoxin by obtaining dust samples from the mother’s bedroom at two home visits, the first at 24–30 weeks of pregnancy and later at 3 months after delivery. The samples will be obtained with a domestic vacuum cleaner coupled to a dust collector provided with a filter (Dustream collector, Indoor biotechnologies). Immediately after being collected, the sample is stored with an ice-pack unit and later stored at − 20 °C until its use. The extraction of allergens and endotoxin from the dust samples will be done according to the previously published protocol [28].

### Ethics and dissemination

The study is performed in accordance to the Helsinki Declaration. The study was approved by the Institutional Review Boards of Medicine Faculty of Pontificia Universidad Católica de Chile, IRB n°170,925,013. The study was also retrospectively registered in the clinicaltrials.gov public database (NCT04186949) on December 5, 2019. Written informed consent is obtained from each pregnant woman and biological father of the baby, which includes the consent for obtaining the child’s data and samples, which is signed by the parents. Results will be disseminated through peer-reviewed manuscripts, conference presentations, and via different media channels to lay public.

### Statistical methods

For descriptive statistics mean ± SD will be used, median and interquartile range for quantitative variables, and percentage with n for categories. Distributions will be verified with a Shapiro Wilk test. Differences in quantifiable biomarkers will be determined by ANOVA or Kruskal Wallis according to data distribution. Spearman (parametric) or Pearson (no parametric) correlations will be applied to determine the association between dependent and independent (maternal and neonatal) variables. Independent variables will be fitted to logistic and lineal regression models to evaluate interactions as well as to identify confounding factors. For in vitro studies, comparisons between two or more groups will be performed by means of Student’s unpaired t-test and analysis of variance (ANOVA), respectively. If the ANOVA demonstrates a significant interaction between variables, post hoc analyses were performed by Dunns when comparing selected groups and multiple-comparison Bonferroni correction test. Comparison of curves and maximal responses under different conditions will be analyzed by ANOVA. All the analyses will be carried out with the statistical software Graphpad Prism or STATA considering a *p* < 0.05 as cut-off for statistical significance.

## Discussion

We expect that the ARIES birth cohort will provide crucial findings to further unveil how allergies and asthma develop in children, considering the complex interaction of biological, social, and environmental factors. In addition, we will obtain valuable information on the incidence and the timing of onset of allergies and asthma in this study population. We thereafter project contributing with key findings that may aid in the search for primary and secondary prevention measures against allergies and asthma in high risk populations.

Like other cohort studies, our study has strengths and limitations. Among the strengths, the ARIES birth cohort study is designed to evaluate how genetic and environmental factors during ‘the first 1000 days’ affect the development of allergies and asthma in children. This cohort study involves multiple disciplines, combining the evaluation of maternal conditions, newborn immune phenotype and epigenetic cues, maternal and neonatal microbiota, genotyping, and the study of environmental factors that can influence the development of allergy and asthma in the child. Multiple biological and environmental samples and measurements are longitudinally being collected and stored. Finally, ARIES is the first birth cohort to study the origins of allergy and asthma in Chile, a country undergoing a rapid epidemiological transition, and it is one of the few birth cohorts’ studies in Latin American population.

Among the limitations of our study, Chile has varying climates and ethnical backgrounds in the north, south and central regions of the country. This study is being done in the central regions only, which could not be representative of all the Chilean population. In addition to this, due to the characteristics of the population attended at the recruitment center, ARIES families mostly have a middle to high socioeconomic status, limiting our potential to evaluate allergies and asthma in low income and vulnerable populations.

## Data Availability

The results of the analysis and data will be disseminated through peer-reviewed manuscripts, conference presentations, and via different media channels to lay public.

## Abbreviations

AD: Atopic dermatitis
FA: Food allergy
AR: Allergic rhinitis
IgE: Immunoglobulin E
IL: Interleukin
ILC2: Type 2 innate lymphoid cells
Th2: T helper 2
GWAS: Genome-Wide Association Studies
DNA: Deoxyribonucleic acid
RNA: Ribonucleic acid
ID number: Identification number
REDCap: Research Electronic Data Capture
EPDS: Edinburgh Postnatal Depression Scale
PSS-14: Perceived Stress Scale
EDTA: Ethylenediaminetetraacetic acid
CBMC: Cord blood mononuclear cells
PBMC: Peripheral blood mononuclear cell
PM: Particulate matter
CO: Carbon monoxide
NO_2_: Nitrogen dioxide
O_2_: Oxygen
SO_2_: Sulphur dioxide
O_3_: Ozone
IRB: Institutional review board
SD: Standard deviation
